# Supplementary material for: Medium-scale integrated circuits based on p-type 2D semiconducting MoTe2
Source: Nat Commun. 2026 Mar 23;17:4320. doi: 10.1038/s41467-026-70992-1 (PMC13172523; doi:10.1038/s41467-026-70992-1)
Supplement: Supplementary file 1 — Supplementary Information [file 41467_2026_70992_MOESM1_ESM.pdf]

Medium-scale integrated circuits based on p-type 2D semiconducting $MoTe_2$

Hui Wang[1], Zebang Luo[1], Biyuan Zheng[1], Zilan Tang[1], Huaidong Ye[2], Yulong Yuan[2], Yizhe Wang[1], Haitao Zhang[1], Qin Shuai[1], Huawei Liu[1], Guangcheng Wu[1], Dong Li[1], Li Xiang[1], Anlian Pan[1,3]**

[1] Key Laboratory for Micro-Nano Physics and Technology of Hunan Province, Hunan Institute of Optoelectronic Integration, College of Materials Science and Engineering, Hunan University, Changsha, 410082, China

[2] Hunan Institute of Advanced Sensing and Information Technology, Xiangtan University, Xiangtan 411105, China

[3] School of Physics and Electronics, Hunan Normal University, Changsha 410081, China

*Email: xiangli93@hnu.edu.cn; anlian.pan@hnu.edu.cn

Supplementary Materials

Supplementary Note

Supplementary Note 1. The contact resistance was analyzed using the Y-function method.

Generally, the total resistance between the source and drain terminals is expressed as $R_{total}=R_{ch}+2*R_C$, where R_{ch} denotes the channel resistance and R_C represents the contact resistance.

When a transistor works in linear region, the Y-function is defined as following:

$$Y = \frac{I_{ds}}{\sqrt{g_m}} = \sqrt{V_{ds} * G_m}(V_{gs} - V_{\mathrm{th}})$$

Where V_{th} is threshold voltage and G_m is the transconductance parameter defined as

$$G_m = \mu C_{ox} \frac{W_{ch}}{L_{ch}}$$

Where $\mu, C_{ox} W_{ch}, L_{ch}$ stand for mobility, unit are gate capacitance, channel width and channel length separately. The relationship between Y and V_{gs} with different L_{ch} is plotted in Supplementary Figure 15 The G_m can be extracted from slope V_{gs}~Y curve, and naturally R_C can be derived from following equation.

$$2R_C = R_{total} - R_{ch} = \frac{V_{ds}}{I_{ds}} - \frac{1}{G_m(V_{gs} - V_{th})}$$

Supplementary Figure

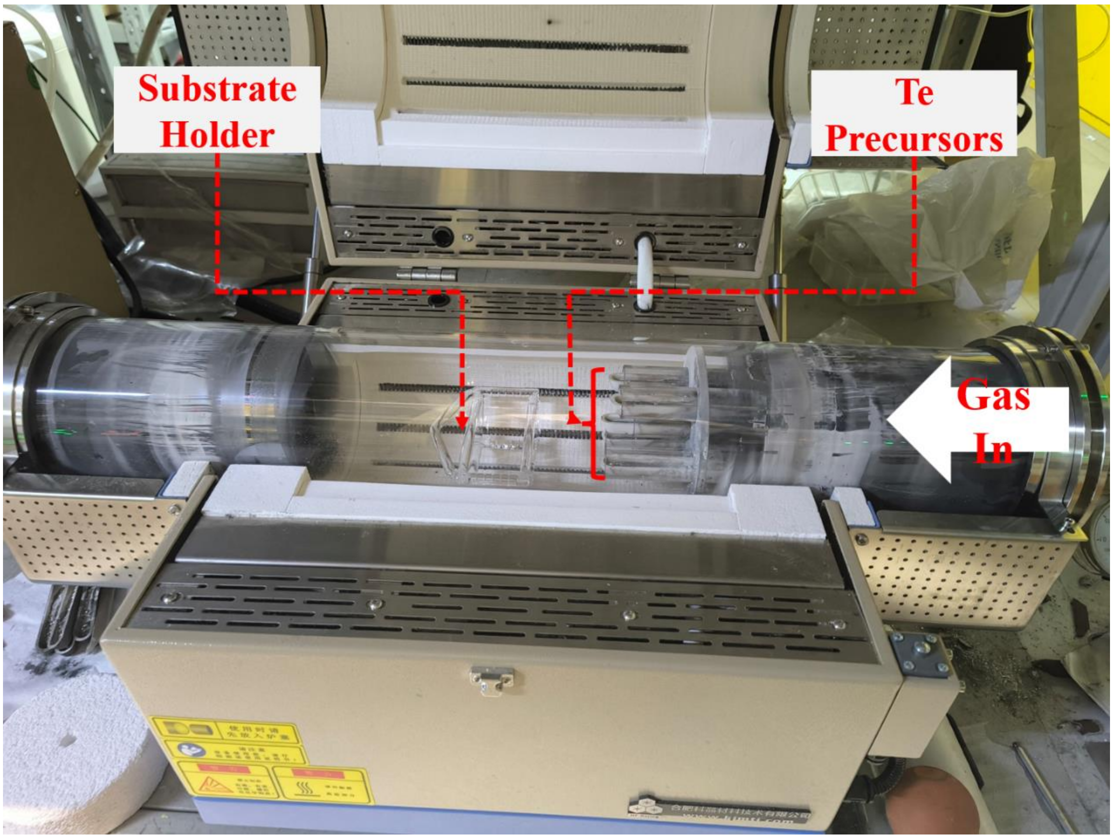

Supplementary Figure 1. Optical image and set-ups of custom-engineered 4-inch $MoTe_2$ CVD growth system.

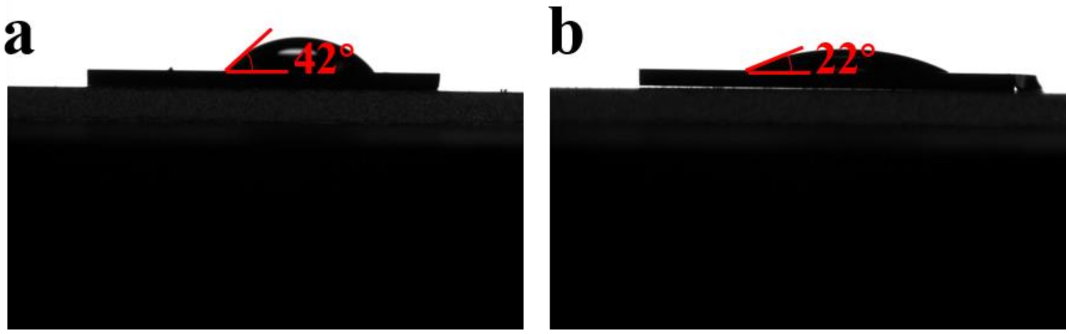

Supplementary Figure 2. The contact angle tests of the Si/SiO_2 substrates before **a** and after **b** high-energy oxygen plasma treatment.

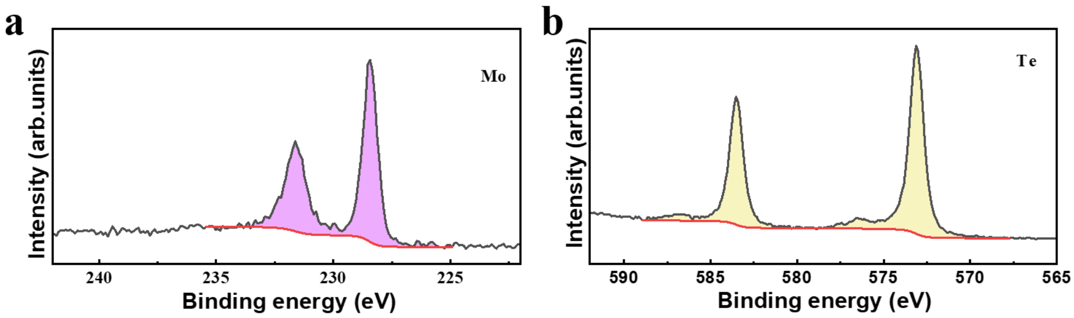

Supplementary Figure 3. High-resolution XPS results of the **a** Mo *3d* and **b** Te *3d* of the 2H-$MoTe_2$.

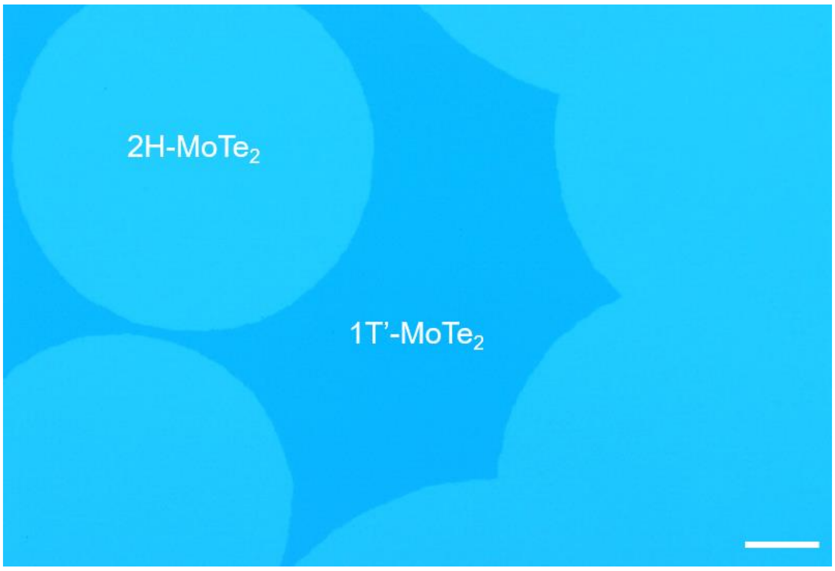

Supplementary Figure 4. Optical image showing the coexistence of 1T′ and 2H phases of $MoTe_2$. Scale bar 200 μm.

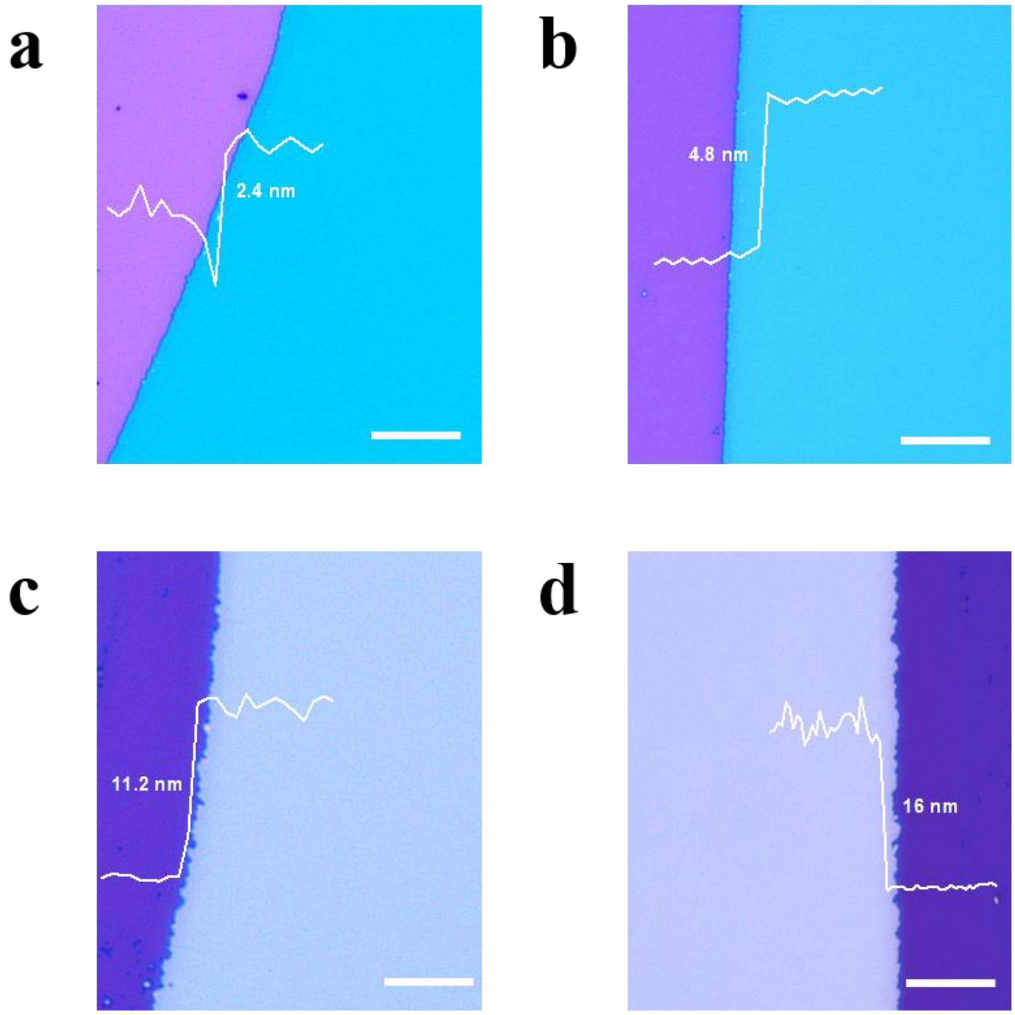

Supplementary Figure 5. Optical images and corresponding AFM height profiles of $MoTe_2$ thin films. **a** showing thickness of 2.4 nm (scale bar 20 μm), **b** showing thickness of 4.8 nm (scale bar 20 μm), **c** showing thickness of 11.2 nm (scale bar 20 μm), **d** showing thickness of 16 nm (scale bar 20 μm).

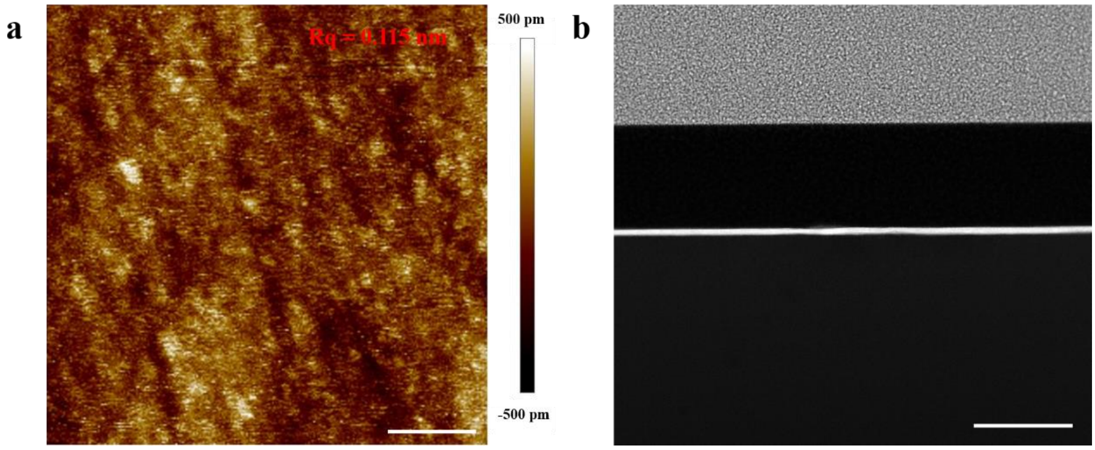

Supplementary Figure 6. a AFM surface topography images of $MoTe_2$ thin films, **b** TEM cross-section of $MoTe_2$ with SiO_2 in the substrate. The image shows a very flat and smooth surface. Scale bar 100 nm.

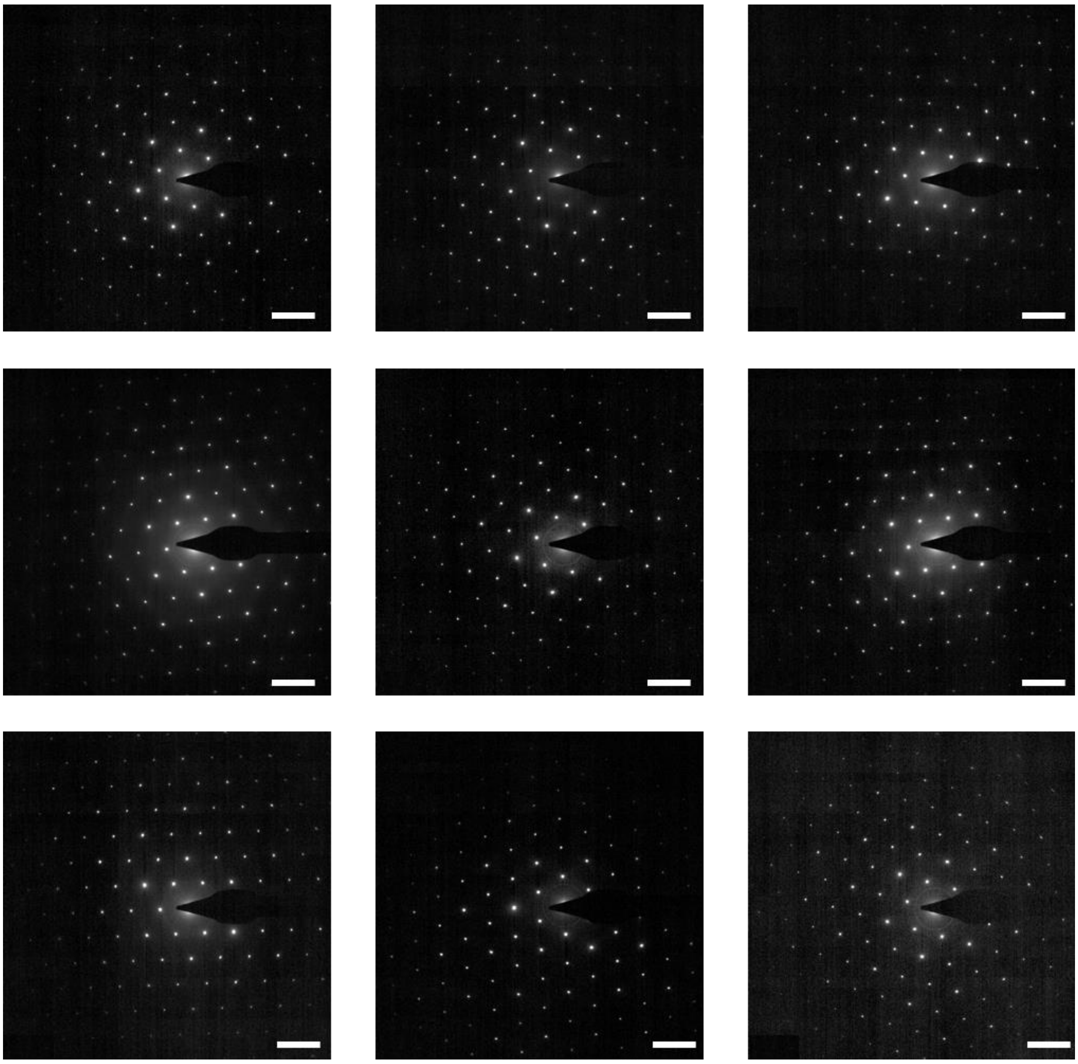

Supplementary Figure 7. The figures show SAED patterns selected from different regions of $MoTe_2$ thin films grown on 4-inch wafers. Scale bar 5 nm.

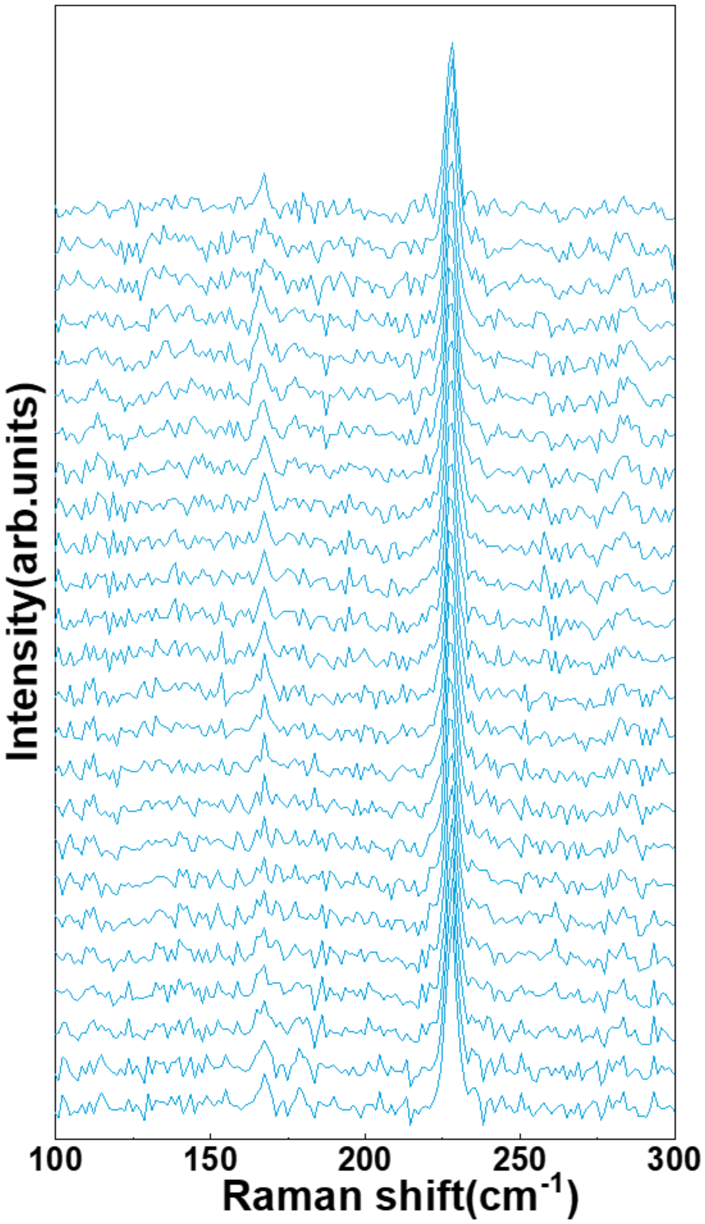

Supplementary Figure 8. A size of Raman spectral of the 25 different sampling points on the four-inch $MoTe_2$ wafer.

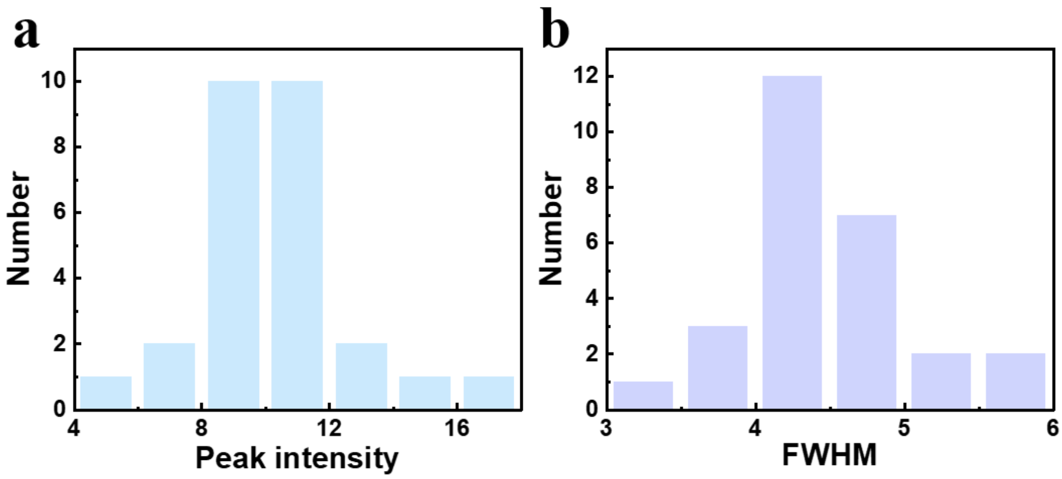

Supplementary Figure 9. The statistical histogram of **a** E_{2g}^{1} peak intensity and **b** FWHM of the 25 sampling points on the 4-inch 2H-$MoTe_2$ wafer.

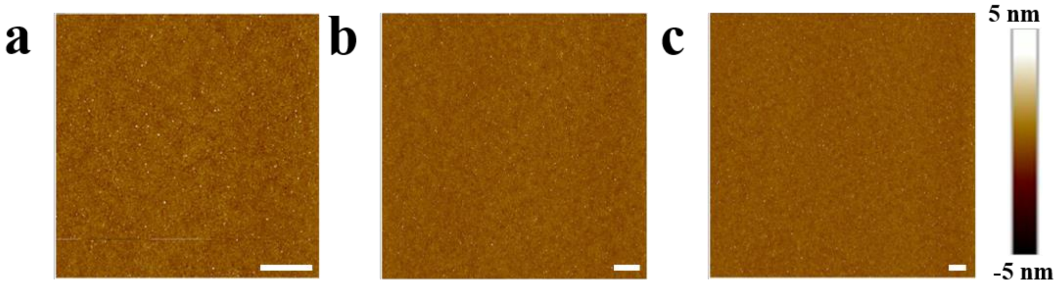

Supplementary Figure 10. AFM sampling was performed on $MoTe_2$ films grown on 4-inch wafers across a range of 5 µm to 15 µm. a Atomic force microscopy surface topography images of $MoTe_2$ films at the 5 µm scale. **b** Atomic force microscopy surface topography images of $MoTe_2$ films at the 10 µm scale. **c** Atomic force microscopy surface topography images of $MoTe_2$ films at the 15 µm scale. Scale bar 1 µm.

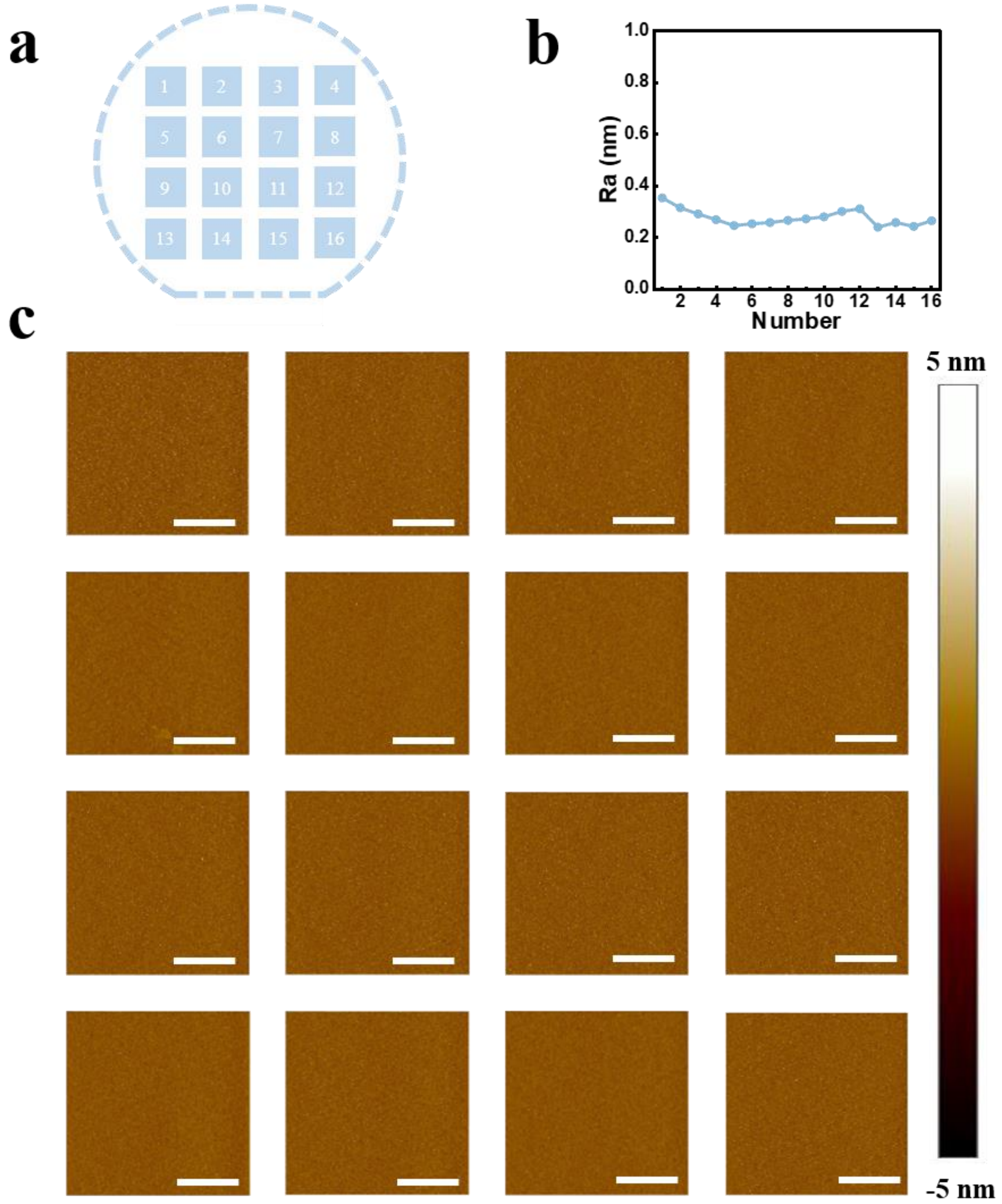

Supplementary Figure 11. a Schematic diagram of a $MoTe_2$ thin film grown on a 4-inch wafer with 16 sampling points., **b** Calculate the Ra values for 16 sampling points. **c** Detailed atomic force microscopy surface topography images of 16 sampling points. Scale bar 5 µm.

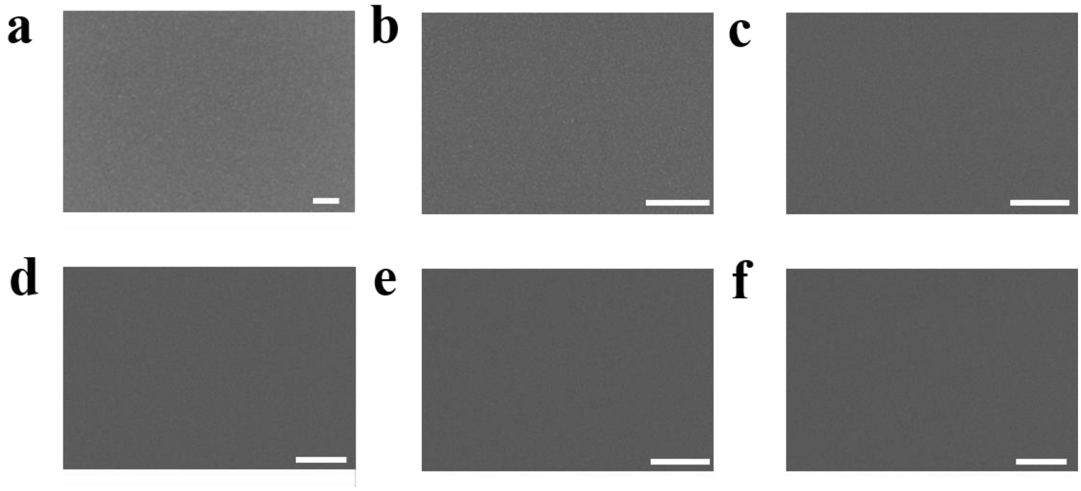

Supplementary Figure 12. Shows the transmission electron microscopy sampling of the $MoTe_2$ films grown on 4-inch wafer at different scanning ranges. a - g Shows the transmission electron microscopy surface morphology of the $MoTe_2$ films at different scales. **a** Scale bar 100 nm. **b** Scale bar 500 nm. **c** Scale bar 1 μm. **d** Scale bar 2 μm. **e** Scale bar 5 μm. **f** Scale bar 20 μm.

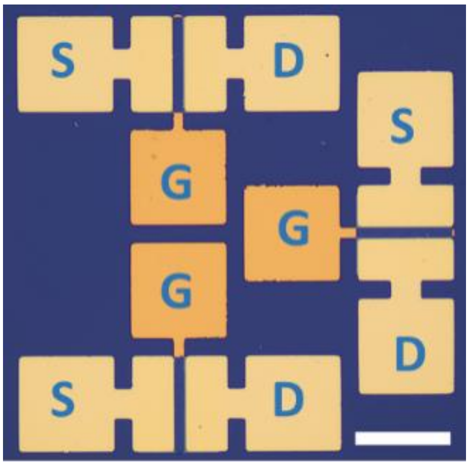

Supplementary Figure 13. the magnified photograph the $MoTe_2$ transistors, where the G, S, and D represent gate, source and drain electrodes, respectively. Scale bar 100 μm.

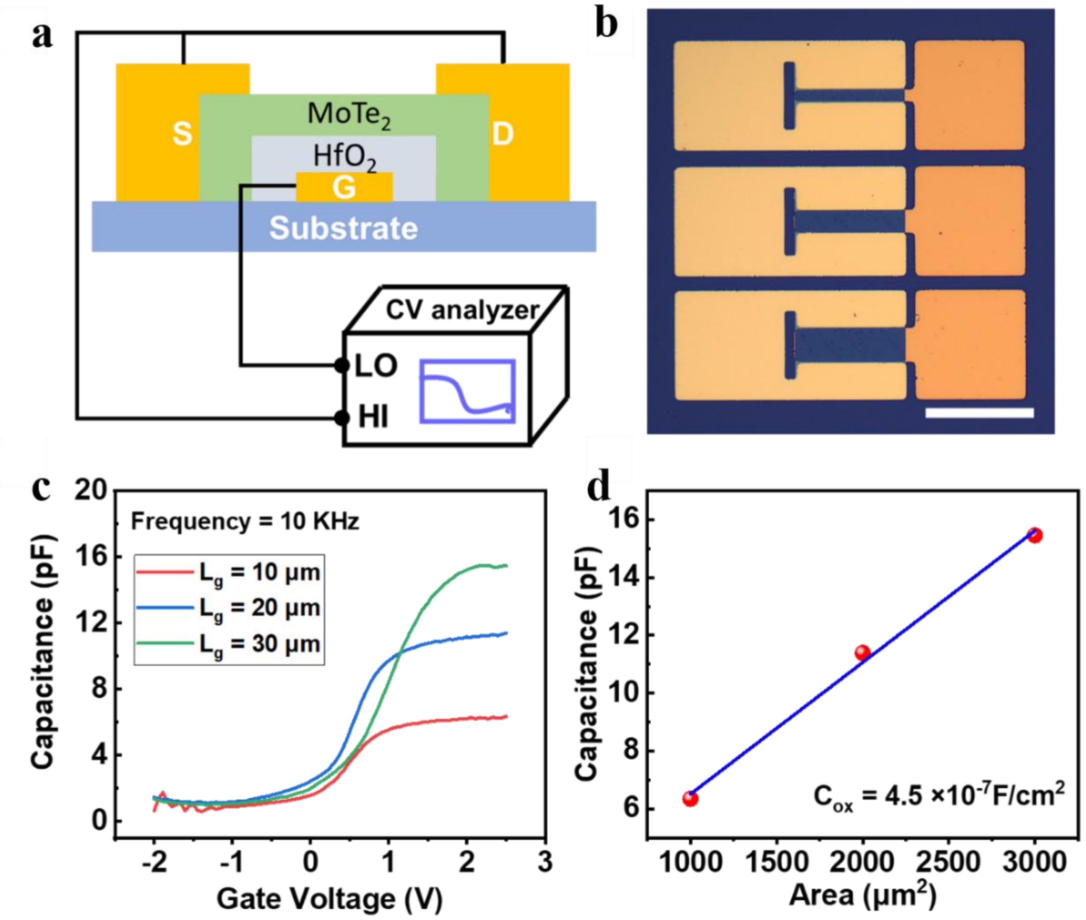

Supplementary Figure 14. C-V measurement setups and characteristics. a Capacitance measurement using the C-V analysis method. **b** Optical image of Metal-oxide-semiconductor capacitors (MOS-CAP) with different areas (scale bar 100 μm) optical image. **c** C-V curves of the MOS-CAPs with different areas measured at low frequency of 10KHz. **d** capacitance as a function of the capacitor area of the MOS-CAPs, where the C_{OX} could be extracted from the slope to be 0.45 μF/cm^2.

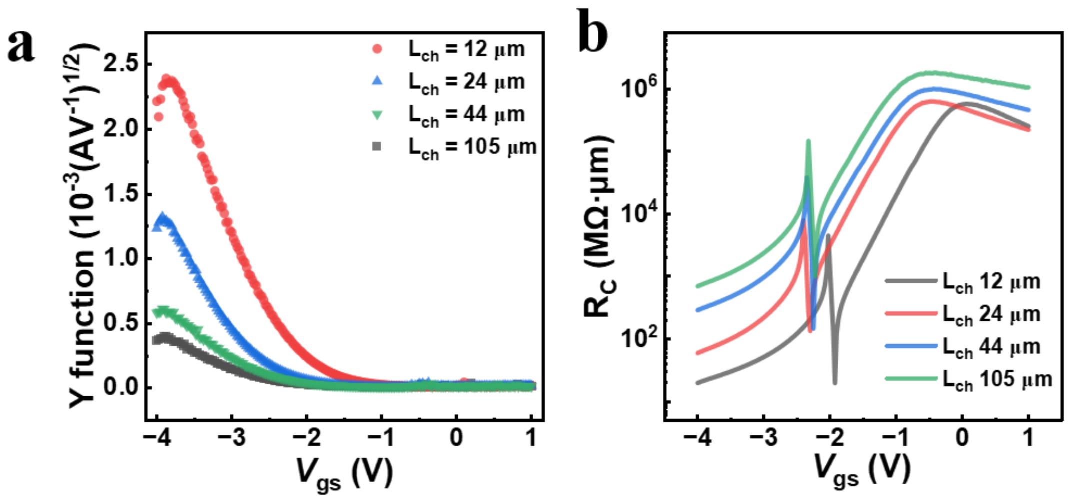

Supplementary Figure 15. The Y function method analysis. a Calculated Y function for the devices shown in a as a function of V_{gs}. **b** The R_c of devices with different L_{ch} (W_{ch} =100μm).

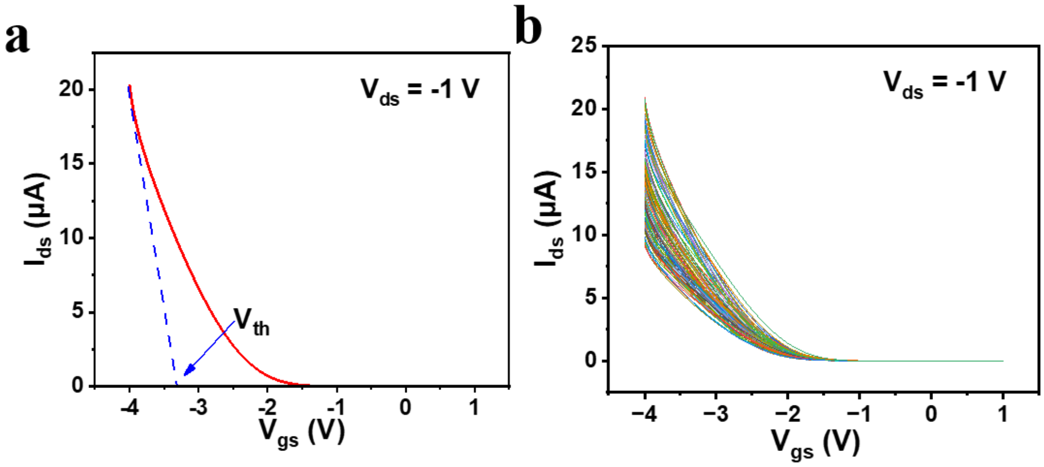

Supplementary Figure 16. Threshold voltage calculation. a The threshold voltage (V_{th}) calculation illustration. The V_{th} is calculated by the intercept of the line extrapolated at the point of maximum transconductance. **b** Linear scale transfer characteristics for 100 TFTs for V_{th} calculation.

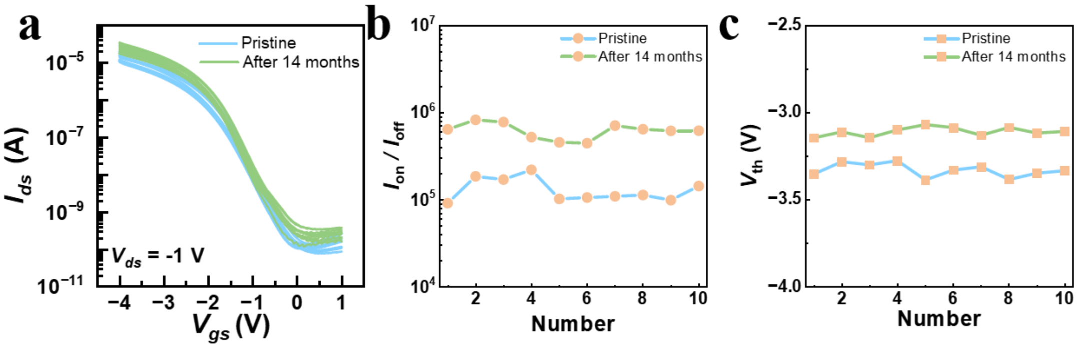

Supplementary Figure 17. a Transfer characteristic of $MoTe_2$ transistors before (blue curves) and after (green curves) 14-month storage. Comparison of the I_{on}/I_{off} **b** and V_{th} **c** curves for $MoTe_2$ transistors before (blue curve) and after (green curve) 14 months of storage.

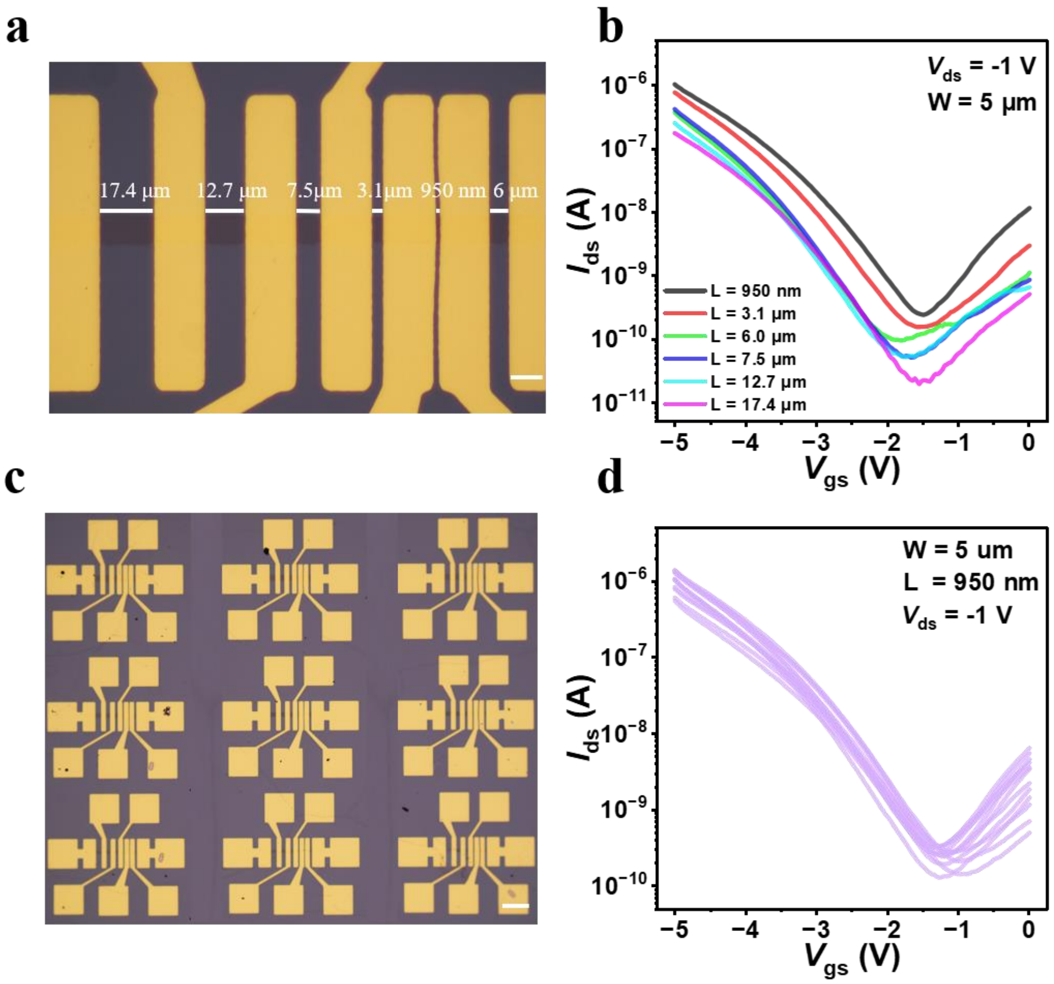

Supplementary Figure 18. $MoTe_2$ transistors with different channel dimensions. a, Optical micrograph of $MoTe_2$ field-effect transistors with varying channel lengths (L). Scale bar 9 µm. b, Transfer characteristics of $MoTe_2$ transistors with different channel lengths measured under identical conditions. c, Optical image of an array of $MoTe_2$ transistors with scaled device dimensions. Scale bar 90 µm. d, Transfer characteristics of 12 representative devices with identical geometry, showing good device-to-device uniformity.

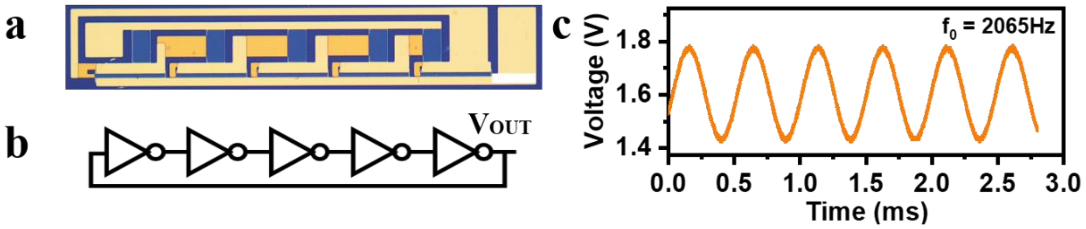

Supplementary Figure 19. a Optical images (scale bar, 100μm), **b** circuit diagrams and **c** input-output characteristics of the 5-stage $MoTe_2$ based ring oscillator.

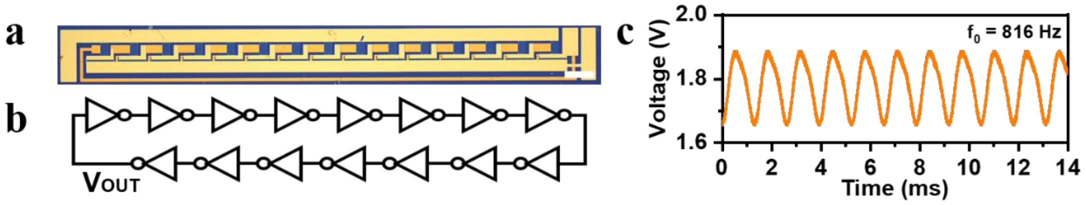

Supplementary Figure 20. a Optical images (scale bar 200 μm), **b** circuit diagrams and **c** input-output characteristics of a 15-stage $MoTe_2$ based ring oscillator.

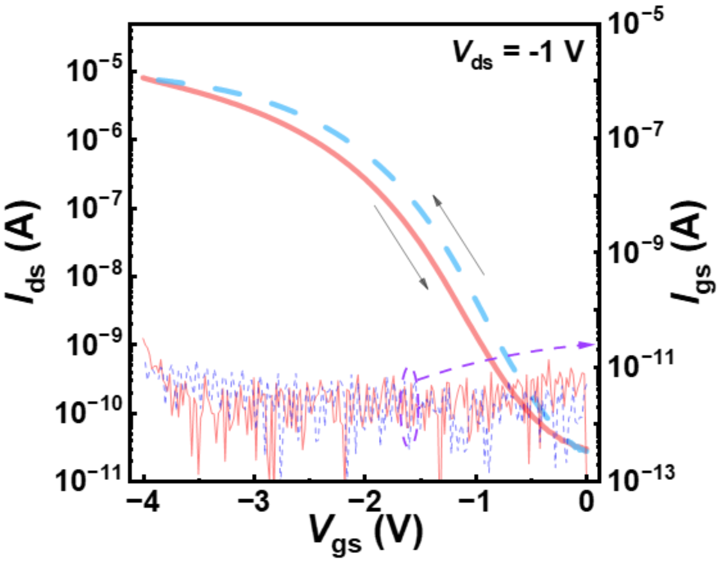

Supplementary Figure 21. Forward and reverse transport in a single $MoTe_2$ transistor.

Supplementary Table 1. Comparison of the integrated circuits utilizing p-type 2D semiconductors.

REFERENCE	MATERIALS	CIRCUIT TYPE*	DEVICE NUMBER	LOGIC DEPTH
Ref. 21	$MoTe_2$	discrete transistors	1	0
Ref. 26	$MoTe_2$	discrete transistors	1	0
Ref. 27	$MoTe_2$	discrete transistors	1	0
Ref. 36	$MoTe_2$	discrete transistors	1	0
Ref. 45	$MoTe_2$	discrete transistors	1	0
Ref. 46	WSe_2	discrete transistors	1	0
Ref. 47	$MoTe_2$	discrete transistors	1	0
Ref. 49	$MoTe_2$	discrete transistors	1	0
Ref. 60	BP	discrete transistors	1	0
Ref. 10	WSe_2	FET array	1	0
Ref. 18	$MoTe_2$	FET array	1	0
Ref. 19	$MoTe_2$	FET array	1	0
Ref. 34	$MoTe_2$	FET array	1	0
Ref. 38	$MoTe_2$	FET array	1	0
Ref. 39	$MoTe_2$	FET array	1	0
Ref. 20	$MoTe_2$	inverter	2	1
Ref. 23	$MoTe_2$	inverter	2	1
Ref. 48	WSe_2	inverter	2	1
Ref. 54	$MoTe_2$	inverter	2	1
Ref. 56	$MoTe_2$	inverter	2	1
Ref. 59	$MoTe_2$	inverter	2	1
Ref. 13	WSe_2	NAND	4	1
Ref. 53	$MoTe_2$	NAND	4	1
Ref. 58	WSe_2	NAND	2	1
Ref. 59	BP	NAND	4	1
Ref. 51	WSe_2	five-stage ring oscillator	10	5
THIS WORK	$MoTe_2$	4-bit full adder	140	13

*Note: Because the reported integrated circuits that utilize p-type 2D semiconductors is very limited, the previous works that only fabricate discrete transistors are included in this comparison.
